# Endophytic Colonization of Onions Induces Resistance Against Viruliferous Thrips and Virus Replication

**DOI:** 10.3389/fpls.2018.01785

**Published:** 2018-12-06

**Authors:** Alexander Mutua Muvea, Sevgan Subramanian, Nguya Kalemba Maniania, Hans-Michael Poehling, Sunday Ekesi, Rainer Meyhöfer

**Affiliations:** ^1^Section of Phytomedicine, Institute of Horticultural Production Systems, Leibniz Universität Hannover, Hanover, Germany; ^2^Plant Health Division, International Centre of Insect Physiology and Ecology, Nairobi, Kenya; ^3^Department of Biological Sciences, Mount Kenya University, Thika, Kenya; ^4^Crop Defenders, Ottawa, ON, Canada

**Keywords:** *Hypocrea lixii*, *Thrips tabaci*, *Iris yellow spot virus*, onions, systemic, host plant resistance, multi-trophic interactions

## Abstract

In agricultural ecosystems, insect pests, pathogens, weather patterns, and reduced soil fertility pose major challenges to crop productivity and are responsible for significant yield losses worldwide. *Iris yellow spot virus* (IYSV) vectored by *Thrips tabaci* Lindeman, is a major hindrance to onion production in eastern Africa. Control measures often rely on insecticides with deleterious effects. Endophytes are one key alternative as they can play important roles in mediating induced systemic resistance. Hence, we examined the potential effect of endophytic fungus *Hypocrea lixii* (F3ST1) on feeding and replication of IYSV on endophyte-colonized (E+) and endophyte-free (E-) onion plants. For more precise assessment, replication was also tested using leaf disk bioassays and individual thrips. The number of feeding punctures was significantly lower in E+ as compared to E- plants. Disease level was significantly lower in E+ as compared to E- plants for four weeks post-exposure to thrips. IYSV replication was reduced by 2.5-fold in endophytic treatment on both whole plant and leaf disk assays. *Thrips tabaci* showed 2 times higher feeding activities on endophyte-free onion leaf disks as compared to the endophyte-inoculated leaf disks. Our results suggest potential utility of the endophytes to reduce feeding damage and virus infection on onion plants. Further studies should be conducted to elucidate the secondary metabolites involved in such endophyte-thrips-virus mediated interaction and determine whether the interactions extend for this and other onion varieties and viruses under field conditions.

## Introduction

Onion, *Allium cepa* L. (Asparagales: Amaryllidaceae), is an important vegetable crop grown for its benefits in subsistence or commercial farming systems worldwide. In Kenya, onions are grown in all counties by both large- and small-scale farmers (Narla et al., 2011). The major factors limiting onion production are pests and diseases (Pappu et al., 2009; Birithia et al., 2011; Gachu et al., 2012). The onion thrips, *Thrips tabaci* Lindeman, is the most economically important pest of onion in Kenya and worldwide (Trdan et al., 2005; Waiganjo et al., 2008). They cause direct damage by feeding on leaf tissues resulting in a reduction of photosynthetic ability and consequently reducing onion bulb size and yield (Rueda et al., 2007; Birithia et al., 2014). Bulb onion yield losses of up to 60% have been reported in Kenya due to thrips damage alone (Waiganjo et al., 2008). Thrips feeding lesions also act as a source of secondary infection by pathogenic fungi and bacteria (McKenzie et al., 1993). Tospovirus, *Iris Yellow Spot Virus* (IYSV) (Bunyaviridae: *Tospovirus*) transmitted by onion thrips is also a major threat to economic production of both bulb and seed onion production globally (Gent et al., 2004, 2006; Pappu et al., 2009) and in eastern Africa (Birithia et al., 2014). Farmers mostly rely on synthetic chemical insecticide applications to manage onion thrips vectoring IYSV (Gachu et al., 2012). However, insecticides can lead to serious environmental hazards in addition to causing pesticide resistance in onion thrips populations (Martin et al., 2003). Hence, to remain effective, control programs have to integrate several disease management tactics that explores next-generation agriculture including the use of beneficial micro-organisms such entomopathogenic fungi which have been reported to play multiple roles in nature (Vega et al., 2008).

*Iris yellow spot virus* is transmitted by *T. tabaci* in a circulative and propagative manner (Whitfield et al., 2005). The virus is acquired by the first or second larval stages and it then multiplies and survives through the later developmental stages (Whitfield et al., 2005; Birithia et al., 2013). Adult thrips emerging from thrips larvae that had acquired IYSV are viruliferous and can transmit the virus. While adults directly feeding on a virus infected plant can acquire the virus, but they cannot transmit it. Strategies that can interrupt this process of acquisition, multiplication and further spread of the virus can lead to development of effective thrips-tospovirus management technologies.

Fungal endophytes are one of such organisms that inhabit and live inside plant tissues without inducing apparent symptoms in their hosts (Rodriguez et al., 2009). In plants primed with endophytes, defense responses are accelerated upon pathogen or insect attack, resulting in enhanced resistance to the attacker (Brotman et al., 2010). Published evidence suggests that endophytic fungi can play symbiotic roles in nature, such as antagonists of plant disease, beneficial rhizosphere colonizers, increased drought tolerance and plant-growth promoters (Vega et al., 2008; Rodriguez et al., 2009; Jaber and Salem, 2014; Jaber and Ownley, 2017). When endophytes colonize plants, they produce enzymes which have the function to suppress plant pathogen activities directly and have the capability of degrading the cell walls of such pathogens (Gao et al., 2010). Emission of secondary metabolites is considered to play an important role during plant defense activities against insects and pathogen attack. Plant colonization by endophytes is also known to influence the population dynamics of insect vectors of diseases. For instance, endophytic isolates of the genus *Neotyphodium* protected meadow ryegrass (*Lolium pretense* = *Festuca pratensis*) from herbivory by bird cherry oat aphid (Lehtonen et al., 2006). A reduction of tunneling in maize by *Ostrinia nubilalis* Hübner (Lepidoptera: Pyralidae) (Bing and Lewis, 1991) and *Sesamia calamistis* Hampson (Lepidoptera: Pyralidae) (Cherry et al., 2004) were attributed to endophytic *Beauveria bassiana* Balsamo (Hypocreales: Clavicipitaceae). Feeding and oviposition were significantly reduced in endophyte–colonized bean plants which in turn affected pupation and adult emergence (Mutune et al., 2016). Similarly, endophytic colonization of banana by *Beauveria bassiana* significantly reduced larval survivorship of banana weevil, *Cosmopolites sordidus* (Coleoptera: Curculionidae), resulting in 42–87% reduction in plant damage (Akello et al., 2008).

Several fungal isolates have been reported to colonize onion plants and confer them protection against thrips through reduced feeding and oviposition resulting in reduced population (Muvea et al., 2014). Further, these authors demonstrated that colonization of onion plants by endophytic fungus, *Hypocrea lixii* F3ST1 triggered antixenotic repellence of *T. tabaci* that impacts their biology (Muvea et al., 2015). On Faba beans Akutse et al. (2013), whilst using the same fungus, reported reduced longevity of progeny, number of pupae and adult longevity of leaf miner flies. Evidence indicates that endophytic fungi can provide plants with protection against plant pathogens (Lehtonen et al., 2006; Ownley et al., 2010; Jaber and Salem, 2014). For instance, *Piriformospora indica* (Hymenomycetes: Basidiomycota), an endophytic root-colonizing fungal species has been shown to repress *Pepino mosaic virus*, which is found widely in tomato greenhouses in many parts of the world, especially at high light intensities (Fakhro et al., 2010).

The mechanism of increased pathogen tolerance in endophyte-inoculated plants is largely speculative, but it is hypothesized that secondary compounds produced by endophytes may play a partial role in this phenomenon (Yue et al., 2000). Mechanisms implicated in plant-endophyte-virus interactions on induced resistance to viral infection may include inhibition of viral multiplication or accumulation (Loebenstein, 1972). The changes in host plant properties can affect viral diseases both directly through host metabolites and indirectly via effects of plant quality on insect vectors transmitting the viruses. Endophytic systemic colonization through intercellular spaces and vascular xylem elements can inhibit or interfere with the systemic movement of plant viruses from cell to cell, which eventually results in delayed multiplication in inoculated plants (Loebenstein, 1972; Martelli, 1980). A case study evaluating the transmission and multiplication of *zucchini yellow mosaic virus* (ZYMV) found lower virus titer levels on endophyte-colonized plants with *Beauveria bassiana* isolates as compared to the endophyte-free plants (Jaber and Salem, 2014). Lehtonen et al. (2006) reported a lower percentage of *barley yellow dwarf virus* (BYDV) infections in endophyte-colonized meadow ryegrass (*Lolium pretense*) compared to endophyte-free plants, indicating that endophyte colonization can protect plants from virus infections and eventual multiplication. Indeed, endophyte-mediated resistance could also impact on the replication of IYSV, in addition to the induced systemic resistance to its vector, *Thrips tabaci* as reported earlier (Muvea et al., 2014, 2015). Extending these findings, in the present study, we hypothesized that endophytic colonization of onion plants by *H*. *lixii* would induce resistance against viruliferous thrips damage and will impact on the virus replication on the insects. This is with a view to obtaining a potential environmentally friendly biocontrol agent in the management of IYSV in onion.

## Materials and Methods

### Insect Rearing

Initial cultures of *T. tabaci* were field-collected from onion plants at the International Centre of Insect Physiology and Ecology (*icipe*) organic farm, Duduville, Nairobi, Kenya. Thrips were reared on snow peas pods, *Pisum sativum* L. (Fabales: Fabaceae), for over 35 generations in ventilated plastic jars at the *icipe’*s insectary at 25 ± 1^o^C, 50–60% relative humidity (RH), 12 h L: 12 h D photoperiod. Adults for the experiment were allowed to lay eggs on snow pea pods for 3 days and were then removed. Neonate first instars (≤ 8 h-old) were used in the subsequent experiments.

### Fungal Isolate

Endophytic fungi *Hypocrea lixii* F3ST1, isolated from the aboveground parts of maize, obtained from tropical highland region in Kenya was selected for this study based on its antagonistic effects against thrips (Muvea et al., 2014, 2015). Conidia were obtained from two-week old cultures grown on Potato Dextrose Agra (PDA) plates. The conidia were harvested by scraping the surface of sporulating cultures with a sterile scalpel. The harvested conidia were then placed in a universal bottle with 10 ml sterile distilled water containing 0.05% triton X-100 and vortexed for 5 min to produce homogenous conidial suspension. The conidial concentration was determined using a Neubauer hemocytometer. The conidial concentration was adjusted to 1 × 10^8^ conidia ml^-1^ through dilution prior to inoculation of seeds. To assess the viability of the conidia, 100 μL of conidial suspension was inoculated to the surface of four fresh plates of PDA. Two sterile microscope cover slips were placed on top of the agar in each plate before incubation. The inoculated plates were incubated for 24 h at 20°C. The percentage conidial germination was assessed by counting the number of germinated conidia out of 100 in one randomly selected field.

### Seed Inoculation and Endophyte Colonization

Seeds of onions (var. Red Creole, East Africa Seed Co., Ltd., Tanzania) were surface-sterilized in 70% ethanol and then immersed in 2% NaOCl for 2 and 3 min, respectively. The seeds were finally rinsed three times using sterile distilled water to ensure that the seed surface was sterilized free of epiphytes. To confirm the efficacy of the surface sterilization, 100 μl of the last rinse water was spread onto four PDA plates and incubated at 20°C for 14 days. The absence of fungal growth on the medium confirmed the reliability of the sterilization procedure (Schulz and Boyle, 2005). The seeds were then placed on sterile filter paper to dry for 20 min and subdivided into two equal portions one for inoculation and the other to serve as the control. Surface-sterilized seeds were soaked in a conidial suspension of 1 × 10^8^ conidia ml^-1^ for 10 h. In the control, seeds were soaked in sterile distilled water containing 0.05% Triton X 100. The inoculated seeds were air dried on a sterile paper towel for 20 min and then transferred to plastic pots (8 cm diameter × 7.5 cm height) containing planting substrate. The substrate was a mixture of red soil and livestock manure in a 5:1 ratio, sterilized in an autoclave for 2 h at 121°C, and allowed to cool down to ambient temperature before being used. Four seeds per pot were sown 1 cm below the surface of the substrate and maintained at room temperature (∼25°C and 60% RH) in a screen house. Endophytic colonization of the inoculated plants was confirmed using the technique described by Muvea et al. (2014). Plants were randomly selected and carefully removed from the pots 50 days after inoculation and the roots washed with running tap water. Leaves, stems and roots of seedlings were cut into different sections. Five randomly selected leaf, stem and root sections from each plant were surface-sterilized as described above. The different plant parts were then aseptically cut into ∼1cm pieces before placing the pieces 4 cm apart from each other, on PDA plates amended with a 0.05% solution of antibiotic (streptomycin sulfate salt). Plates were incubated at 25 ± 2°C for 10 days, after which the presence of endophyte was determined. Prior to incubation of the different plant parts, the last rinse water was also plated out to assess the effectiveness of the surface sterilization procedure as described above. The colonization of the different plant parts was recorded by counting the number of pieces that showed the inoculated fungal growth. Only the presence of the endophyte used for inoculation was scored. After testing for colonization, the remaining seedlings were thinned to one per pot and watered once per day.

### Inoculum Preparation and Virus Transmission Through Mechanical Inoculation

The inoculum was prepared by grinding freshly harvested and virus-infected onion leaves with phosphate buffer at PH 7.4 (1:5 weight/volume). A sterilized mortar and pestle were used for grinding. Forthy four potted whole plant without endophyte and virus were arranged in four groups and placed in separate cages were used for the experiment. Freshly emerging leaves were dusted with carborundum (silicon carbide, 400–600 mesh) to increase infection by providing minute wounds for entry of the virus particles. The inoculum was gently rubbed on the leaves as a source of infection. Simultaneously but in separate cages, the same number of potted plants was used for thrips transmission experiment. However, ten viruliferous thrips were released per plant for this experiment. After 2 weeks, the presence of the virus was tested using IYSV-specific DAS-ELISA (Agdia Biofords) for up to four times.

### Acquisition and Transmission of IYSV by *Thrips tabaci*

Virus transmission using *T. tabaci* was adopted for this study because mechanical inoculation attempted as described was not successful and this was also reported previously (Bulajić, 2008; Srinivasan et al., 2010; Naveed and Pappu, 2012). A cohort of 500 first instar (≤ 8 h-old) *T. tabaci* obtained from *icipe*’s insectary were allowed to acquire virus by feeding on IYSV-infected *Allium cepa* var. Red creole (plants maintained at *icipe* as virus inoculum source) for an acquisition access period (AAP) of 16 h. The virus infection in the plants leaves (1 g) used for virus acquisition was confirmed using IYSV-specific ELISA Flashkit (Agdia Biofords, Netherlands) (Birithia et al., 2013). Thrips were then transferred and reared on snow pea pods until adults emerged.

### Monitoring Replication of IYSV in the Thrips Vector

Virus replication in their vectors has important epidemiological implications as it allows the vector to remain infective throughout their life stages for transmission of viruses (Ullman et al., 1993). In vector thrips of IYSV, *T*. *tabaci* has shown competence in virus load increase from acquisition as 8 h old larva up to adult stage (Birithia et al., 2013). Moreover, studies on its non-structural (NS) proteins have been reported for their specificity for monitoring IYSV replication (Birithia et al., 2013). The expression of the NSs protein in thrips was monitored by direct antigen-coated (DAC) ELISA (Srinivasan et al., 2012). DAC-ELISA was carried out as described by Wijkamp et al. (1993) with slight modifications. Two cohorts of 200 first instar (≤ 8 h-old) *T*. *tabaci* were given an AAP of 16 h on IYSV-infected and non-infected *Allium cepa* var. red creole. They were transferred thereafter to non-infected snow pea pods placed in Petri dishes (8 mm diameter). From each cohort five insects were sampled at 0, 4, 12, 24, 48 and 72 h post-acquisition (h.p.a.) and at pre-pupal and 2-day-old adult stages. Thrips were placed in 1.5 mL Eppendorf tubes containing 100 μL coating buffer (1.59 g Na_2_CO_3_, 2.93 g NaHCO_3_ in 1 L distilled water, pH 9.6) and stored at -20°C until analysis. Thrips were triturated in the buffer with a sterile blunt-end plastic pestle. The suspension was transferred to flat bottom ELISA plates and incubated for 12 h at 4°C. Plates were washed three times with phosphate-buffered saline (PBS) containing 0.05% Tweed 20 (PBS-T) and blocked with 200 μL 1% bovine serum albumen (BSA) for 2 h at 37°C. After draining the plates and washing once with PBS-T, 200 μL polyclonal anti-NSs antibody diluted (1:4000) in antibody dilution buffer {0.2% BSA, 2% polyvinylpyrrolidone (PVP) mol. wt. 40 000, 0.02% sodium azide, pH 7.4} was added to the wells, and the plates were incubated at 37°C for 2 h. Plates were washed three times with PBS-T and 200 μL goat anti-rabbit IgG-alkaline phosphatase (universal conjugate, Agdia Biofords) in antibody dilution buffer (1:500) was added to each well and the plates were incubated at 37°C for 2 h. Absorbance values were measured at 405 nm 1 h after the addition of substrate (0.5 mg mL^-1^ disodium q-nitrophenyl phosphate in 1 M diethanolamine, 0.5 mM MgCl_2_, 0.02% sodium azide) on an ELISA plate reader (Biotek-Epoch).

### Impact of Endophyte Colonization of Onion Plants on Virus Transmission by Thrips

Four 10-weeks-old plants two each from Endophyte-colonized (E+) and Endophyte-free (E-) plants were placed in an equidistant and alternating pattern in 44 thrips-proof cages (40 cm × 30 cm), cages herein referred to as replicates. Virus transmission was performed by releasing 20 viruliferous adults thrips per cage and allowed to feed for 48 h after which all the thrips were removed from the plants using a Carmel brush. The cages were then randomly divided into four groups (Group 1, 2, 3, and 4) with each group comprising of 11 cages (each cage consists of four plants two each from E+ and E-) whereby for instance, group one represented samples for week one in that order. This was considered important to enable assessment of virus replication over time. To exclude bias in our results on virus transmission on E+ and E- treatments, samples (1 g leaf) for the test were cut from sections of the plant with visible feeding punctures. This was necessary because random selection would imply that E+ will have less titer as feeding punctures are positively correlated with virus transmission (Jiang et al., 2000). Samples were obtained after 2 weeks post thrips exposure. Feeding damage was assessed in both treatments from the 44 replicates by counting the number of punctures from four leaf sections each 3cm long per treatment. The excised leaf sections were then tested for virus using IYSV-specific double-antibody sandwich enzyme linked immunosorbent assay (DAS-ELISA, Agdia Biofords). Non-colonized plants (without endophyte and virus) were tested simultaneously for baseline titers.

To obtain a more precise assessment, transmission of IYSV was additionally analyzed using leaf disk assays and individual thrips. This was done to confine the thrips to a food source because previous reports suggest that thrips feeding on whole plants can be repelled leading to reduced feeding and eventual virus replication (Muvea et al., 2014, 2015). Twenty two potted onion plants each for E+ and E- raised in separate cages were used for the leaf disk experiment. A single leaf disk was obtained from the second middle leaf of each plant. The assay was done by allowing viruliferous individual adult thrips which were reared and infected as described earlier to feed on 2 cm^2^ onion leaf disks placed in Petri dishes (9 cm diameter). The top of the Petri dishes was sealed with Parafilm to prevent escape of thrips. A single adult thrips was allowed to feed on each leaf disk for 48 h period. Feeding damage on the leaf disks was confirmed by counting the number of feeding punctures. The same leaf disks were used to test for the presence of the virus as described earlier. ELISA readings were considered positive when the absorption (OD = 405 nm) of the sample wells was at least two times greater than the mean absorption of negative control samples.

### Statistical Analysis

All data were checked for normality and homogeneity of variance before analysis. One-way Analysis of variance (ANOVA) was performed to determine accumulation of NSs protein in thrips and comparisons of means at 95% significance was undertaken with Tukey’s Honestly Significant Difference (HSD) test. Feeding was determined by counting the number of punctures on leaf sections and taking the average per treatment (E+ and E-) for all the 44 replicates before analysis by negative binomial regression using package *MASS* (Venables and Ripley, 2002). The negative binomial distribution was deemed appropriate for this kind of study because of its biological appropriateness and ability to handle over distribution in count data and better goodness of fit measurements (deviance and Pearson chi-square closer to 1) compared with Poisson or Gaussian distributions (Candy 2000). Virus titer levels on whole plants over time was analyzed using repeated measure analysis of variance (ANOVA) and a Bonferroni *post hoc* test using package *multcomp* (Hothorn et al., 2008). Treatments were considered as fixed effects and the cages as replicates. Petri dish experiment analysis was performed using a chi-squared test. *P*-values were based on type III chi-square values in all the analyses. All statistical analyses were performed in R 2.15.3 (R Core Team, 2013).

## Results

### Evaluating Thrips vs. Mechanical Transmission of IYSV

**FIGURE 1:**
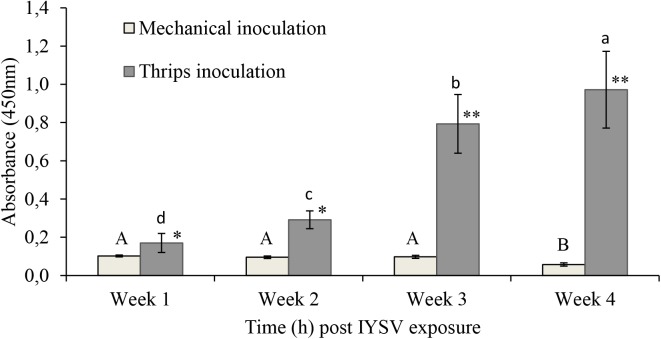
*Iris yellow spot virus* titer levels on onion plants inoculated through mechanical and thrips transmission methods (*n = 11*). Means followed by the same lower or upper case letters indicate no significant differences between different time intervals for IYSV replication through thrips and mechanical transmission, respectively. Asterisks indicate statistically significant differences between treatments at each time point ^∗^*P* < 0.05, ^∗∗^*P* < 0.01.

Biotests on transmission of IYSV using thrips and mechanical modes of transmission showed that the mechanical method of virus transmission is not effective. Hence, the virus transmission method using thrips was selected for this study (Figure 1).

### Endophytic Colonization

In viability tests, conidial germination of F3ST1 before seed inoculation was 90% which was acceptable. The final rinse of water used for surface sterilization of seeds was effective as no sign of fungal growth was observed on the plating media. The average endophytic colonization of onion plant parts was 81, 53.6, and 48.6% for root, stem and leaf sections, respectively.

### Confirmation Test for Acquisition of IYSV by *T*. *tabaci*

**FIGURE 2:**
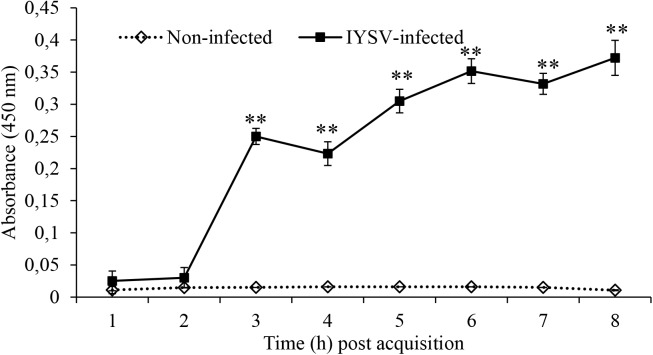
*Iris yellow spot virus* non-structural protein levels in *Thrips tabaci* determined at varying times post-acquisition using specific antibodies. Asterisks indicate a significant difference in NS replication between IYSV-infected and non-infected thrips ^∗^*P* < 0.05, ^∗∗^*P* < 0.01.

Thrips were tested for positive acquisition of IYSV by quantification of NS proteins from larval to adult stages and our results recorded mean titer level of 0.015 at larval and 0.36 at adult stage. Results of the biotest showed that thrips samples tested positive for the virus and there was a significant increase of NS proteins in thrips fed on IYSV-infected plants (*F*_1,78_ = 115.32, *P* < 0.001; Figure 2).

### Effect of Endophytic Colonization on Feeding and IYSV Replication

**FIGURE 3:**
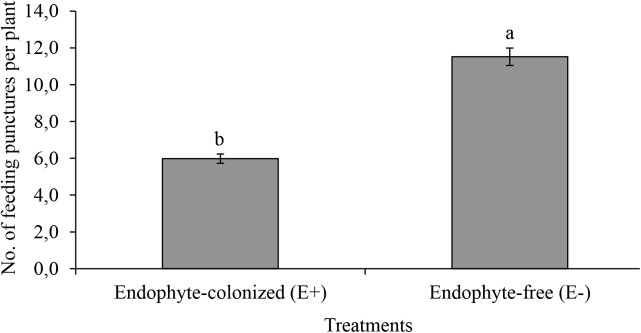
Effect of endophytically colonized onion plants on mean feeding punctures by viruliferous adult *Thrips tabaci*. The figure quantifies mean feeding activity by 20 viruliferous *T*. *tabaci* exposed for 48 h on endophyte-colonized (E+) and endophyte-free (E–) onion plants. Bars indicate means ± SE at 95% CI (*n* = 44). Different letters indicate a significant difference between treatments.

**FIGURE 4:**
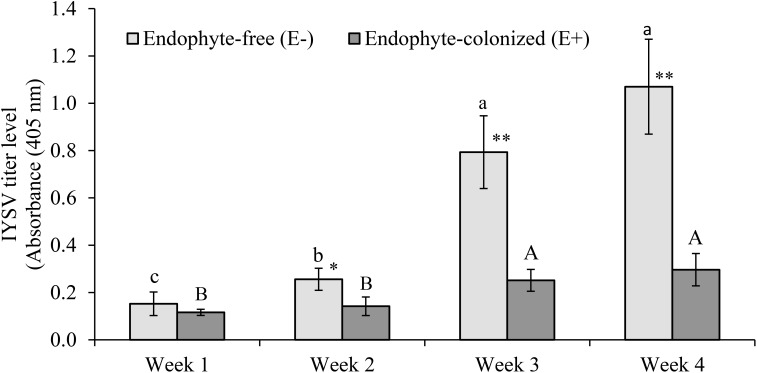
Effect of endophytically colonized onion plants by *Hypocrea lixii* F3ST1 on IYSV replication overtime. Means followed by the same lower or upper case letters indicate no significant differences between different time intervals for IYSV replication on E– and E+, respectively. An evaluation of endophytic fungus for its effect on IYSV transmission by viruliferous thrips after 48 h as measured from samples taken from a whole plant. Means ± (standard error) SE at 95% confidence interval (*n = 11*). Asterisks indicate statistically significant differences between treatments at each time point ^∗^*P* < 0.05, ^∗∗^*P* < 0.01.

The number of feeding punctures were significantly lower in endophyte-inoculated plants as compared to the control treatment (χ2 = 19.67, *df* = 1, *P* < 0.001, *n* = 44; Figure 3). There was approximately a 2-fold reduction in feeding activities on E+ as compared to E- plants (Figure 3). Since feeding was assessed on random sections of the plant, the data was collected and recorded before using the samples for IYSV test. Replication of IYSV was reduced 2.5-fold on endophytically colonized onion plants (Figure 4). Endophyte-colonized plants showed lower IYSV titer levels of 0.23 ± 0.07 as compared to 0.58 ± 0.11 from the endophyte-free plants (*F* = 5.98; *df* = 1, 10; *P* < 0.001; Figure 4). The effect of time in regard to virus replication was significant for E- plants (*F* = 10.98; *df* = 3, 10; *P* < 0.001). However, there was no significant difference in the level of IYSV replication on E+ plants over time (*F* = 1.02; *df* = 3, 10; *P* = 0.39) (Figure 4). The third and the fourth week of data sampling showed a 3.2 and 3.6-fold increase in IYSV replication in E- compared to E+ plants, respectively (Figure 4). The average ELISA titer value for non-infected controls for four weeks (without endophyte and virus) was 0.11 ± 0.003 which was 2 and 5-fold lower than the readings for E+ and E- plants, respectively.

**FIGURE 5:**
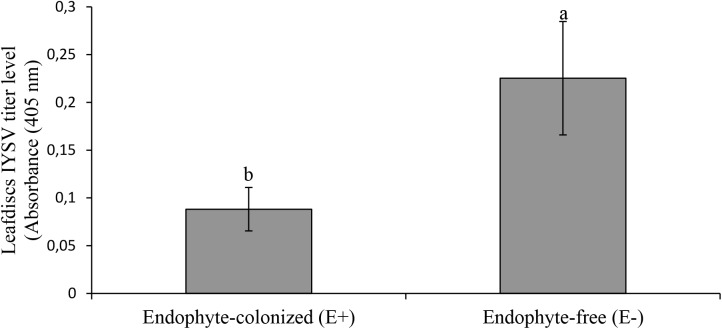
Effect of endophyte-inoculated and endophyte-free onion leaf disks on transmission of IYSV by viruliferous *Thrips tabaci*. Bars indicate average titer levels (±SE) after a 48h feeding period (*n = 22*). Different letters indicate a significant difference between treatments.

**FIGURE 6:**
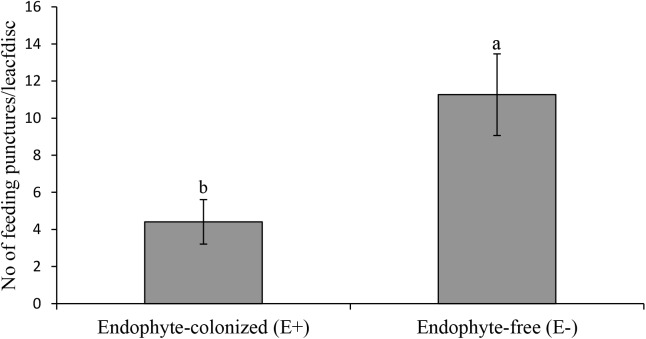
Effect of endophyte-inoculated and endophyte-free onion leaf disks on number of feeding punctures by viruliferous *Thrips tabaci*. Bars indicate average titer levels (± SE) after a 48 h feeding period (*n = 22*). Different letters indicate a significant difference between treatments.

Results from the leaf disk assay showed a 2.5 fold reduction of IYSV replication on endophyte-colonized onion plants (χ2 = 4.65, *df* = 1, *P* = 0.03) (Figure 5). *Thrips tabaci* showed 2.5 fold reduction in feeding activities on endophyte-colonized as compared to the endophyte-free onion leaf disks (Figure 6).

## Discussion

Colonization of the onion plants by fungal endophytes in a previous study by Muvea et al. (2014) was found to significantly reduce feeding, oviposition and survival of onion thrips, *Thrips tabaci.* Among the endophytes tested, *Hypocrea lixii* (F3ST1) was the best performing isolate (Muvea et al., 2014), which was selected for the current study. *H*. *lixii* F3ST1, isolated from above ground parts of maize and sorghum has been reported to be endophytic on several host plants such as maize, onion and bean seedlings with antagonistic properties against pests such as leaf miners and thrips (Akello, 2012; Akutse et al., 2013; Muvea et al., 2014). This study further aimed to assess the poorly understood interaction between the endophyte and the thrips-transmitted virus, IYSV. Optimization of an effective virus transmission protocol is critical in such interaction assays. Comparative evaluation of mechanical and thrips-transmission of IYSV indicated that only thrips-transmission was effective, and therefore was adopted further in the study. This is in line with the previous reports by Bulajić, 2008, Srinivasan et al. (2010), and Naveed and Pappu (2012).

Endophyte-colonization of onion plants reduced feeding activity of viruliferous *T*. *tabaci* by 2-fold as compared to endophyte-free (E-) onion plants. In a previous study using non-viruliferous *T*. *tabaci*, feeding activities were reduced on endophyte-colonized onion plants and it was speculated that antibiosis and/or antixenosis repellency of thrips could have played a key role (Muvea et al., 2014, 2015). Similar observations have also been made in other endophyte-pest interactions such as *Neotyphodium*-aphid interaction in New Zealand tall fescue (Guy and Davis, 2002) and *Hypocrea/Beauveria*-leafminer interaction in Faba bean (Akutse et al., 2013). Overall, our findings highlight the utility of endophytic colonization in reducing thrips feeding damage on onions.

This study further indicates that replication of IYSV in *H. lixii*-colonized onion plants was significantly reduced as compared to endophyte free plants. There was a significant reduction in IYSV titer levels by 4 weeks after virus infection in whole plant and leaf disk assays. In the first week of sampling, difference in titer levels of IYSV between E+ and E- plants were not significant. However, the differences became apparent from the second week onwards and increased up to 2.8-fold in E- plants by the fourth week. The virus replication in the E+ plant was maintained significantly low as compared to the non-colonized controls. This could be explained due to a combination of lower feeding by viruliferous thrips on E+ plants and the reduced ability of the virus to replicate in the E+ plants. Inoculation of *Pinus halepensis* Mill seedlings with fungal endophytes including *Trichoderma* spp. significantly reduced leaf necrosis length caused by a plant pathogen, *Gremmeniella abietina* (Lagerberg) Morelet (Romeralo et al., 2015). Similarly, three endophytic isolates of *Lecanicillium* sp. suppressed production of *Podosphaera fuliginea* (Schlecht.) Pollacci spores, a plant pathogen that causes powdery mildew (Kim et al., 2007). Whereas there are numerous reports on the ability of endophytes to reduce plant pathogens (Ownley et al., 2008), to our knowledge, this is among the first report to reveal fungal endophytes from Ascomycota (*H*. *lixii*) as endophytic colonizers with ability to reduce tospovirus replication. The negative interaction of *H. lixii* to IYSV is closely similar to reports on reduction of incidence and severity of aphid-borne potyvirus, ZYMV in *B. bassiana*-colonized plants as compared to the non-colonized plants (Jaber and Salem, 2014). Our results are also concordant with those reported by Lehtonen et al. (2006) on meadow ryegrass using *Neotyphodium uncinatum* Gams, Petrini & Schmidt, endophytes. The authors reported reduced transmission and replication of aphid-transmitted Luteovirus, BYDV on endophyte-colonized as compared to endophyte-free plants. The authors speculated that the effect on the feeding activities of aphids on endophyte-colonized plants was the reason for the lower virus infection frequency in endophyte-colonized meadow ryegrass.

The mechanism underlying these negative effects on the vectors and the plant virus are not well understood. This could likely be due to endophytic fungi triggering gene expression in defense pathways thus increasing production of plant defensive compounds (Jung et al., 2012; Pieterse et al., 2014). The impact of fungal secondary metabolites produced *in planta* in their endophytic association could also play a role in this interaction. IYSV titre levels were lower in endophyte-colonized plants in spite of leaf samples being picked from plants with signs of feeding damage for both E+ and E-. This further adds to the speculation that a mechanism of resistance could be prevalent and which is an outcome that needs further investigation. Shrivastava et al. (2015) in a recent study reports that co-infection of endophytic *B*. *bassiana* and mycorrhizae in tomato plants can significantly increase terpenoid content in leaves leading to a reduction in foliar feeding by herbivores. Alkaloids released by endophytic fungi have also been reported to possess antiviral activities (Selim et al., 2012). Hence to further understand the mechanism underlying these interactions, analysis of the secondary metabolites and the head space volatiles from endophyte colonized plants is critical. Although our laboratory and screen house experiment results cannot be extrapolated to field conditions and other onion-thrips-variety and plant-pest systems, our results suggest that this fungus may more likely enhance plant resistance to insect feeding and virus transmission when they become endophytic. Various factors in the field such as plant diversity, other insect pests and pathogens and prevailing environmental conditions are likely to influence the multitrophic interaction between the host plant-endophyte- virus-insect vectors which needs to be further investigated.

To conclude, our studies imply that endophytic colonization of onion plants may act as deterrence to feeding damage by viruliferous thrips. Moreover, endophytic colonization enhances the ineffectiveness of viruliferous thrips to transmit IYSV and has negative effects on IYSV replication in the plant. Consequently, endophytic fungi should be considered as an important component which needs to be taken into account when investigating plant-endophyte-insect interactions. This knowledge has clear implications for understanding the epidemiology of insect-transmitted plant diseases and improving their management options under integrated agricultural systems. A better understanding of the interaction between the insect, virus, endophytes and the host plant is important since biochemical and physiological factors may act in combination or independently, resulting in differences in efficacy of selected endophytes on viruliferous insects feeding on different onion varieties.

## Author Contributions

AM, RM, SS, H-MP, SE, and NM conceived and designed the experiments. AM, SS, and NM performed the experiments. AM, SS, and RM analyzed the data. NM, SS, and SE contributed reagents, materials, and analysis tools. AM, NM, SS, H-MP, SE, and RM contributed to the writing of the manuscript. SS, RM, NM, H-MP, SE, and RM obtained funding. All authors contributed to revisions and commented on previous versions of the manuscript.

## Conflict of Interest Statement

The authors declare that the research was conducted in the absence of any commercial or financial relationships that could be construed as a potential conflict of interest.
